# Leave entitlements, time off work and the household financial impacts of quarantine compliance during an H1N1 outbreak

**DOI:** 10.1186/1471-2334-12-311

**Published:** 2012-11-20

**Authors:** Anne M Kavanagh, Kate E Mason, Rebecca J Bentley, David M Studdert, Jodie McVernon, James E Fielding, Sylvia Petrony, Lyle Gurrin, Anthony D LaMontagne

**Affiliations:** 1 Centre for Women’s Health, Gender and Society, Melbourne School of Population Health, The University of Melbourne, Melbourne, Victoria, Australia; 2 Centre for Health Policy, Programs and Economics, Melbourne School of Population Health, The University of Melbourne, Melbourne, Victoria, Australia; 3 Vaccine & Immunisation Research Group, Murdoch Children’s Research Institute and Melbourne School of Population Health, The University of Melbourne, Melbourne, Victoria, Australia; 4 Victorian Infectious Diseases Reference Laboratory, North Melbourne, Victoria, Australia; 5 National Centre for Epidemiology and Population Health, The Australian National University, Canberra, Australian Capital Territory, Australia; 6 Victorian Government Department of Health, Melbourne, Victoria, Australia; 7 Centre for Molecular, Environmental, Genetic and Analytic (MEGA) Epidemiology, Melbourne School of Population Health, The University of Melbourne, Melbourne, Victoria, Australia; 8 McCaughey Centre, Melbourne School of Population Health, The University of Melbourne, Melbourne, Victoria, Australia

## Abstract

**Background:**

The Australian state of Victoria, with 5.2 million residents, enforced home quarantine during a H1N1 pandemic in 2009. The strategy was targeted at school children. The objective of this study was to investigate the extent to which parents’ access to paid sick leave or paid carer’s leave was associated with (a) time taken off work to care for quarantined children, (b) household finances, and (c) compliance with quarantine recommendations.

**Methods:**

We conducted an online and telephone survey of households recruited through 33 schools (85% of eligible schools), received 314 responses (27%), and analysed the subsample of 133 households in which all resident parents were employed.

**Results:**

In 52% of households, parents took time off work to care for quarantined children. Households in which no resident parent had access to leave appeared to be less likely to take time off work (42% vs 58%, p=0.08) although this difference had only borderline significance. Among parents who did take time off work, those in households without access to leave were more likely to lose pay (73% vs 21%, p<0.001). Of the 26 households in which a parent lost pay due to taking time off work, 42% experienced further financial consequences such as being unable to pay a bill. Access to leave did not predict compliance with quarantine recommendations.

**Conclusions:**

Future pandemic plans should consider the economic costs borne by households and options for compensating quarantined families for income losses.

## Background

Social distancing and quarantine measures were central to Australia’s response to the outbreak of pandemic (H1N1) 2009 influenza (influenza A(H1N1)pdm09 (REF WHO)). Established community transmission of the novel virus was first confirmed in Victoria, Australia’s second largest state with 5.5 million residents. The majority of infections in the early weeks of the outbreak occurred among school-aged children. This high paediatric case proportion prompted the Victorian government to close classrooms and entire schools, introduce voluntary home quarantine for many children and their families, and recommend additional social distancing.

A previous study found that non-pandemic influenza in school-aged children causes significant disruption to usual household activities, including lost work days for parents [1]. Home quarantine during the 2009 influenza outbreak in Australia may have accentuated such difficulties for two reasons. First, the length of time for which quarantine was recommended was up to seven days, which is considerably longer than usual school absences. Second, the recommendation that quarantined children not have exposure to non-household members restricted childcare options.

Paid leave entitlements are an important buffer against ‘shocks’ to childcare arrangements; a US study found that parents with access to paid leave are more likely to stay home to care for sick children than parents without such entitlements [2]. When presented with a hypothetical scenario of a pandemic, employees in insecure jobs that lacked leave entitlements reported that they would be less likely to comply with social distancing measures [3], and indeed a recent study in the US found that work-related barriers to imposing social distance was associated with increased incidence of influenza-like illness during the H1N1 outbreak [4]. One-quarter of working Australians do not have access to paid leave [5], one of the highest levels in the OECD. This raises questions about their capacity to have taken time off work during the 2009 Victorian influenza outbreak, the impact on household finances if they did, and their ability to facilitate full compliance with the quarantine restrictions imposed on their children. A study that preceded the 2009 outbreak, suggested that up to a third of Australians may experience financial difficulties if quarantined for longer than two weeks [6].

We conducted a cross-sectional survey of parents of children who were asked to go into home quarantine during the initial stages of the influenza A(H1N1)pdm09 outbreak in Victoria, which unfolded between 20 May and 3 June 2009. In earlier publications from this study we examined compliance with the quarantine measures, and the information that affected households received about these measures [7,8]. In most of the affected households, compliance with quarantine recommendations would have necessitated the children being cared for by a parent in the home. This analysis focuses on the subset of households in which all resident parents were employed during the quarantine period and no parent was him/herself quarantined. Compared to households in which one or more parents had access to paid leave, we hypothesised that households without this access would: (i) be less likely to have a parent take time off work; (ii) be at greater risk of adverse financial consequences (because some would take leave regardless); and (iii) have poorer compliance with quarantine recommendations.

## Methods

### Study environment

The first Australian case of influenza A(H1N1)pdm09 was identified on 8 May 2009. Two weeks later, Victoria’s first case was identified – a nine-year-old boy who had recently returned from the United States [9]. In the ensuing 12-day period, ‘contain’ pandemic response measures [10] including case isolation, voluntary home quarantine and school closure were implemented, in an effort to prevent wider community spread of the imported virus.

During this response phase, cases and their immediate family members and close contacts were asked to go into home quarantine [11]. Quarantined persons were expected to have no contact with non-household members and were treated with oseltamivir for ten days. Cases were asked to stay in quarantine for seven days after the onset of symptoms. Contacts—defined as individuals who spent more than four hours in the same room as a confirmed case, or were within one metre of a confirmed case for more than 15 minutes—were asked to stay in home quarantine for seven days from last date of exposure to the case (Department of Health Victoria quarantine guidelines, 4 June 2009).

The trigger for closure of mainstream schools was two or more confirmed cases in separate classes. Where a single case was identified, only the class or immediate teaching group was closed. However, only cases and fellow students who met the definition of contacts were placed in home quarantine; other students were asked to limit their outside activities (Department of Health Victoria quarantine guidelines, 4 June 2009). At special developmental schools a single confirmed case triggered home quarantine for the entire student body.

### Sample

The target population for this study was households in which a child had been asked to go into home quarantine during the outbreak, from schools affected by class closures during the outbreak. We identified eligible households through schools. During the outbreak, the Victorian Departments of Education and Early Child Development (DEECD) and Health (DoH) and the Catholic Education Office were actively involved in visiting schools, identifying cases and determining the need for quarantine. Each of these agencies held separate but incomplete information on quarantine activities in schools. After pooling this information, we approached principals at 82 schools and posed two eligibility questions: did the school have (i) classes closed during the ‘contain’ phase of the outbreak? and (ii) children who were asked to go into home quarantine?

The study’s original sample size calculations were based on preliminary estimates from the Victorian Departments of Health and Education about the number of eligible schools affected by closures, the number of children in those schools and the number of households affected. Of 82 schools identified, six did not provide information to allow us to assess their eligibility, and of the schools that did provide requisite information, only 39 met the eligibility criteria. This reduced the number of in-scope households significantly below what was anticipated. Of the eligible schools, 33 agreed to facilitate the conduct of the survey (school participation rate was 85%).

We worked with staff at participating schools to identify 1,188 families with children who went into quarantine. School staff used enrolment records, class lists and documentation of which classes and students had been asked to enter quarantine in order to identify these families. We advised and guided school staff regarding the assembly and review of this information but had no contact with data identifying students or families.

The study was approved by the University of Melbourne ethics committee (0932293) and the DEECD and the Catholic Education Office granted us permission to approach schools to conduct the survey.

### Survey administration

We tested a draft version of the survey instrument for comprehension, length and ease of administration with three participants from eligible schools, and made minor modifications based on their feedback. Due to the need to administer the survey as soon as possible after the school closures occurred, so as to reduce recall bias and maximise participation, more extensive testing was not feasible. The finalised survey was administered during November and December 2009. School staff mailed letters to the parents in eligible families inviting them to participate. The letter presented two options: an internet address at which parents could complete the questionnaire online and a telephone number to ring to complete it via a Computer Assisted Telephone Interview (CATI). The survey was offered in English only. The letter also included a unique identification number which enabled access to the website and CATI. This number allowed us to identify the school(s) and home class(es) associated with each survey response, but revealed no other identifying information. A copy of the CATI questionnaire is included in an online Additional file 1: Appendix.

School staff mailed two reminder letters. To boost response rates and recognise the effort of participating families and schools we contributed $AU20 to the school for the purchase of educational resources for each completed questionnaire and all families received a movie voucher valued at AUS$10.30 with the second reminder letter.

**Figure 1:**
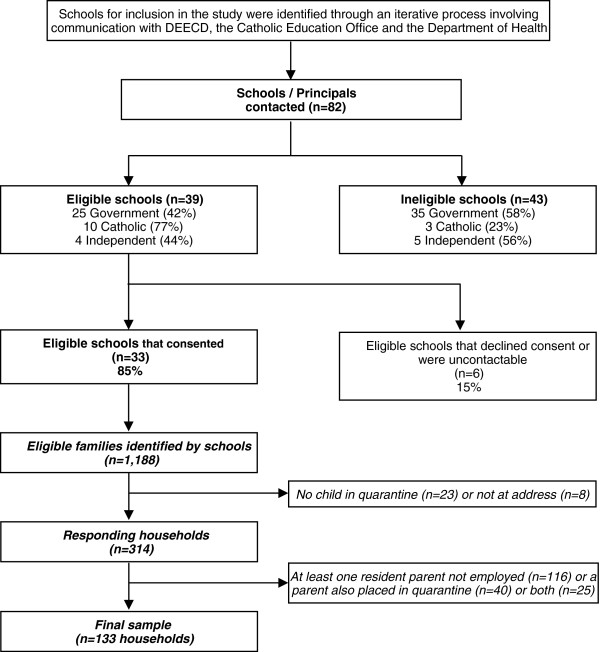
Recruitment of sample of parents whose school children were recommended to go into home quarantine (May 22nd until June 2nd, 2009), and restriction of final sample for this analysis to households in which all resident parents were employed during the quarantine period and in which no parent had been asked to stay in voluntary home quarantine.

Eight letters were returned-to-sender and 23 parents responded indicating that they did not have a school-aged child who had been placed in home quarantine. This left an in-scope sample of 1,157. We received 314 responses, yielding a household participation rate of 27% (see Figure 1).

### Variables

#### Care arrangements during quarantine

For each child in quarantine, responding parents were asked to indicate who (e.g. parent, older sibling, grandparent, paid carer) provided any care for the child during school hours in the quarantine period. We then categorised households according to whether a parent provided any such care for any quarantined child.

#### Time off work and financial consequences

In households reporting that a parent had provided care for their quarantined children during school hours we asked if they took any time off work to do so and, if they did, whether this time off work was paid or unpaid. For those who took unpaid time off work, we asked them whether they had to borrow money, had difficulty paying a bill, mortgage or rent, or experienced other financial problems as a result.

#### Access to leave

We defined parental leave entitlements according to whether each employed parent reported having access to paid sick leave or paid carer’s leave. This definition did not include annual leave. Parents who did not have paid sick or carer’s leave entitlements, or were unaware of their leave entitlements, were classified as not having access to leave. Households were then classified as having access to leave if any parent had leave entitlements, or not having access to leave if no parent did.

#### Compliance with quarantine recommendations

A household’s compliance with quarantine recommendations was assessed using the following criteria:

1. All quarantined members of the household stayed at home for most of each day.

2. Quarantined children did not mix with children from another household for 15 minutes or more.

3. No adults from other households visited the home for 15 minutes or more.

4. No quarantined household members visited public places being utilised by lots of other people (excluding visits to health practitioners).

5. Childcare was provided only by adults living in the household.

We constructed an overall measure of compliance distinguishing households that met all the criteria from those that did not.

### Statistical analyses

Analysis was restricted to the 133 households in which all resident parents were employed during the quarantine period and in which no parent had been asked to stay in voluntary home quarantine (see Figure 1); for the rest of the households surveyed we assumed that non-working parents would have been able to provide childcare. According to whether a household had access to leave, we calculated the proportion of households in which (i) quarantined children were cared for by a parent during school hours; (ii) a parent took time off work to provide this care; and (iii) a parent lost pay as a consequence of taking time off work. We report p values from Pearson’s χ^2^ tests for differences. We also describe the financial consequences of losing pay.

We used logistic regression to quantify the association (estimating odds ratios and 95% confidence intervals) between access to leave or taking time off work and compliance across all five indicators as well as the overall measure. We tested whether the estimates changed by more than 20% with the addition of two potential confounders – highest level of parent education and parental structure of household (single/couple). Addition of the covariates led to substantial attenuation of estimates (>20% change) in four of the six models assessing access to leave and compliance. Accordingly, all models reported in this paper were adjusted for these confounders. Robust standard errors were used to accommodate the fact that data from households were clustered within schools. All analyses were conducted in Stata 11.0 (College Station, TX, USA: StataCorp LP).

## Results

Table 1 outlines the demographic characteristics and leave and childcare arrangements of households in the study sample. In 82% (109/133) of households a parent cared for their quarantined child during school hours and in 52% (69/133) a parent took time off work to care for their child. In 39% (52/133) of households no parent had access to paid sick or carer’s leave during the quarantine period, despite the sample being restricted to only those households in which all parents were in the paid workforce.

**Table 1:** Characteristics of sample (n = 133)

	**no. (%)**
Parental structure in household	
Single parent	15 (11.3)
Highest level of parental education	
University bachelor degree or higher	84 (63.1)
Childcare arrangements during quarantine	
A parent cared for quarantined children during school hours on ≥1 day	109 (82.0)
Time off work	
A parent took time off work to care for quarantined children	69 (51.9)
Access to leave	
No parent in household had access to paid sick/carer’s leave	52 (39.1)

Of the 133 households in the analysis, only eight (6%) contained somebody who had been diagnosed with influenza A(H1N1)pdm09.

### Leave entitlements and care arrangements during quarantine

The proportion of households in which a parent looked after quarantined children on at least one day during the quarantine period did not differ significantly between households with and without access to paid leave (83% vs 81%, p=0.78).

### Leave entitlements and time taken off work

**Figure 2:**
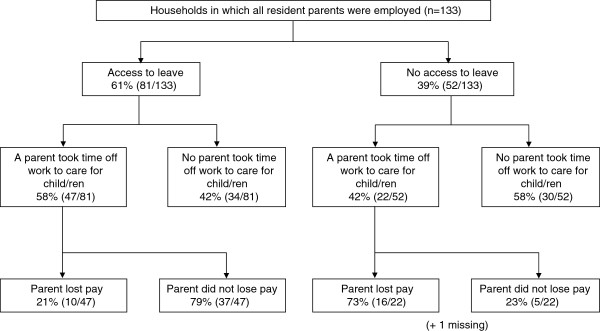
Diagrammatic representation of leave entitlements, time taken off work and financial consequences during the quarantine period (n = 133).

A larger proportion of households with access to leave had a parent who took time off work to care for a child (58% (47/81) vs 42% (22/52) but this difference did not reach statistical significance (p=0.08). Figure 2 shows in greater detail the time taken off work and financial consequences of households in the sample, according to whether or not households had access to paid leave.

### Financial consequences

Across the sample, thirty-eight per cent of households (26/69) lost pay as a result of taking time off work to care for quarantined children. Loss of pay was more frequent in households that did not have access to leave (73% vs 21%, p<0.001) (Figure 2, bottom row).

Of the 26 households in which a parent lost pay (independent of access to leave), 42% (11/26) had at least one other financial problem as a result. Twenty-three per cent (6/26) had difficulty paying a bill, 15% (4/26) had difficulty paying the mortgage or rent, 8% (2/26) had to borrow money and 19% (5/26) had other financial problems.

### Compliance with quarantine recommendations

Half of all households were fully compliant with quarantine recommendations. Compared to households without access to sick leave or carer’s leave, households with access to leave appeared more likely to have all quarantined members stay at home for most of the time on all days during the quarantine period (88% compared with 75%), However, the association was not statistically significant in multivariable analyses that adjusted for parental structure and parental education (OR=2.07; 95% CI 0.82 to 5.23; p=0.12). Further, there was no evidence to support associations between leave entitlements and any other of the four measures of compliance (see Table 2).

**Table 2:** **Logistic regression analysis of access to leave**, **time taken off work and compliance with quarantine recommendations** (**n** = **133 households**)*

	**Stayed at home all days**		**No mixing with children**		**No mixing with adults**		**No trips**		**Childcare by household members only**		**Full compliance**	
	**%**	**OR (95%CI)**	**%**	**OR (95%CI)**	**%**	**OR (95%CI)**	**%**	**OR (95%CI)**	**%**	**OR (95%CI)**	**%**	**OR (95%CI)**
No access to leave	75.0	1.00	75.0	1.00	61.5	1.00	88.5	1.00	88.5	1.00	46.2	1.00
Access to leave	87.7	2.07 (0.82-5.23)	80.3	1.24 (0.63- 2.45)	62.7	0.99 (0.54-1.82)	92.6	1.61 (0.49-5.28)	87.7	0.92 (0.41-2.05)	51.9	1.20 (0.62-2.34)
Did not take time off work	76.6	1.00	71.9	1.00	64.1	1.00	84.4	1.00	82.8	1.00	46.9	1.00
Took time off work	88.4	2.47 (1.17-5.22)	84.1	2.10 (0.71-6.19)	60.9	0.88 (0.32-2.40)	97.1	7.20 (1.42- 36.51)	92.8	2.69 (0.60-12.07)	52.2	1.27 (0.61-2.67)

*Adjusted for highest level of parental education and household structure (single versus two parent).

Turning to the relationship between taking time off and quarantine compliance (independent of access to leave), quarantined members of households in which a parent took time off work were less likely to make trips to populated public spaces during the quarantine period (97% vs 84%) and these households were more likely to have all quarantined members stay at home for most of the time on all days during the quarantine period (88% vs 77%). After adjustment for parental education and parental structure of households, taking time off work was associated with over double the odds of staying at home on all days (OR 2.47, 95% CI 1.17–5.22, p=0.02) and seven times the odds of not making trips outside the home (OR 7.20, 95% CI 1.42–36.51, p=0.02). Taking time off work was not, however, associated with full compliance (see Table 2).

## Discussion

During Victoria’s outbreak of influenza A(H1N1)pdm09 in 2009, parents appeared to be somewhat more likely to take time off work to care for their children when a parent in the household had access to paid sick or carer’s leave, compared to households without access to leave, but there is insufficient statistical evidence to reject the null hypothesis of no difference. Taking time off work was associated with two indicators of compliance with quarantine recommendations: quarantined children staying home for most of the time on all days and not making trips to populated places. However, this study found no evidence that access to leave, per se, was associated with overall compliance with quarantine recommendations. On the other hand, lack of access to leave had measurable negative impacts on families. In households without this benefit available, nearly three-quarters had a parent who lost pay, compared to one in five households with leave, and nearly 40% of households that lost pay experienced further financial difficulties as a consequence.

The chief explanation for the lack of association between access to leave and compliance with quarantine appears to be that families frequently chose to follow public health recommendations even when that meant absorbing the collateral employment-related effects due to inadequate leave entitlements: in 42% of households that did not have access to leave, a parent still took time off work to care for the quarantined child. This behavioural response is particularly selfless in light of the fact that financial consequences are borne privately whereas the benefits of home quarantine and social distancing measures accrue to the community in the form of reduced risks of transmission. While some of this behaviour may have been driven by the need to care for sick children, there were no confirmed influenza A(H1N1)pdm09 diagnoses in the vast majority (94%) of households in our sample. This suggests that, absent the strict quarantine restrictions, other childcare options may well have been attractive to parents to enable them to attend work during the period of school closure. Twenty-two per cent of households where a parent did have access to leave still lost pay as a result of taking time off work. The likely explanation is that, because leave was defined at a household level, a parent without access to leave was the one who took time off work.

Our study is the first we know of to have considered the effect of parental leave entitlements on quarantine compliance during the 2009 outbreak of influenza A(H1N1)pdm09. In Western Australian school closures during this outbreak, a parent took time off work in 45% of households [12] — a similar finding to our study. However the Western Australian study did not examine whether time taken off work influenced compliance or whether taking leave had a financial impact. Our finding contrasts with findings from studies in the US, both hypothetical and real, which have suggested a lack of access to paid sick leave is a barrier to social distancing [3,4].

The study had several limitations. First, despite beginning with a sample frame consisting of all households in Victoria affected by school closures, our relatively small analytic sample meant the study was underpowered to detect differences unless they were large. A good example of this is the relationship between parents’ access to leave and their decision to take time off work to care for their children; the difference in proportions was substantial (16 percentage points) but did not attain statistical significance, likely due to the small sample size. Second, our response rate was not high, despite the use of incentives to boost participation rates. This has implications both for power and the risk of Type II errors. Nonetheless, the response rate is comparable to that achieved in other similar school-based studies of pandemic influenza in the US and England [13-15] and our study had the advantage of covering a larger number of affected schools than most other studies. As we showed in an earlier publication from this study, we received a disproportionately low level of response from less advantaged schools, reducing the generalisability of our findings and potentially biasing our results [8]. It could be expected that non-responding households were less likely to have access to paid leave and may have experienced greater financial consequences, resulting in the estimates presented in this paper being conservative. Unfortunately, the survey had to be administered through schools due to privacy constraints, and we are therefore not able to characterize non-respondents in more detail.

The study was also limited by the fact that the survey was administered several months after the school closures occurred, and all information was obtained via self-report, introducing the possibility of recall bias. In some cases, parents were reporting on behaviours of their children at times when parents may not have been present.

All pandemic plans must balance the likely benefits and social and economic costs of implementing social distancing measures. Characterising the costs incurred by families during quarantine and social distancing of school children during Victoria’s 2009 outbreak of pandemic influenza contributes to the evidence base for future assessment of the costs and benefits of these containment strategies. Models of pandemic influenza have shown that the greatest impact of school closure on transmission is observed when closures are widespread, initiated early, and sustained beyond the epidemic peak [16-18]. In Victoria, school closure was localised, short-lived (often less than 7 days) and reactively initiated following case identification.

In households where parents are forced to take leave from work due to public health emergencies, foregoing wages is a high price to pay for honouring a public duty. Employers should be encouraged to provide flexible working arrangements, such as allowing employees to work from home or to make up hours at a later date. Setting aside the question of whether access to paid sick leave should be available to all workers, there are strong ethical arguments [19] and community support [20] for the provision of compensation to individuals who experience loss of income as a result of public health measures such as quarantine. Policy initiatives along these lines are not unprecedented: several countries affected by the SARS outbreak introduced some form of compensation for affected households [21]. In Australia, this might involve government and employers sharing the costs of compensating quarantined employees. This could operate similarly to the current legislated arrangements for jury service, whereby employers are required to release employees for jury service and pay them the difference between the set jury payment provided by the courts and what they would have received as earnings for that period had they not been on jury service [22].

## Conclusions

Our findings emphasise the importance of bolstering quarantine measures that target children in public health emergencies with a supportive environment in which working parents are able to respond appropriately. We show that in the absence of this environment the social and economic costs borne by families during public health emergencies are non-trivial and unevenly distributed across the affected population. Planning for future pandemics should involve a careful weighing of these costs against the demonstrated effectiveness of any quarantine or social distancing strategies employed. Finally, if home quarantine of school children is implemented, the public and private sector should work to alleviate financial burdens that arise from loss of pay and financial hardship due to the need for affected parents to take time off work.

## Competing interests

The authors declare that they have no competing interests.

## Authors’ contributions

AK conceived of the study and drafted the manuscript; KM developed the analytic plan and conducted the analyses; RB contributed to the conception of the study and design and advised on analysis; JM, JF, AL and DS contributed to the conception of the study and design; LG advised on the analysis and SP contributed to the design of the survey and was responsible for its implementation. All authors contributed to the drafting of the manuscript and have read and approved the final manuscript.

## Pre-publication history

The pre-publication history for this paper can be accessed here:

http://www.biomedcentral.com/1471-2334/12/311/prepub

## Supplementary Material

Additional file 1 0593 H1N1 Swine Flu and Schools Research Project. Click here for file
