# Supplementary material for: Leave entitlements, time off work and the household financial impacts of quarantine compliance during an H1N1 outbreak
Source: BMC Infect Dis. 2012 Nov 20;12:311. doi: 10.1186/1471-2334-12-311 (PMC3533824; doi:10.1186/1471-2334-12-311)
Supplement: Additional file 1 — 0593 H1N1 Swine Flu and Schools Research Project. [file 1471-2334-12-311-S1.pdf]

0593 H1N1 FINAL Questionnaire v8 (clean)

Screening

[MANDATORY]
S0 To ensure we are talking to the right person, are you the parent of a child who is a student at ^name of school^?

1. Yes
2. No **[TERMINATE 1]**

[MANDATORY]
S1 In this survey, 'quarantine' is used to describe situations where people were asked to stay at home to prevent the spread of swine flu between the 22nd May and 3rd June 2009.

Was your child/children asked to go into quarantine between 22nd May and 3rd June 2009?

1. Yes
2. No **[TERMINATE 2]**

Module A; family

[ASK ALL] [MANDATORY]

A0 How many people, including yourself, live in your household?

1. One, just myself **[TERMINATE 3]**
2. Two
3. Three
4. Four
5. Five.
6. Six
7. Seven
8. Eight
9. Nine or More

[ASK ALL] [MANDATORY]

A1 In this section we ask some questions about your family. These answers will help us understand how quarantine and class closures may affect different types of families.
We would like to start by asking the age, gender and relationship to you of all the persons who usually lived in your household during the quarantine period (22nd May to 3rd June).
Please start by providing your age and gender.

[IF A0=9 display: ^Although your household as 9 or more members we would only like to ask you about 8 of them, in the 8 you choose to talk to us about, please ensure you include yourself and your Spouse/Partner and children aged 5 to 18 years old, giving priority to the ones attending ^school name^**.]**

[SHOW AS MANY ROW AS A0 ANSWER, Max 8 rows]

	a **[grid/OPEN NUM]**	b **[GRID/SR]**		c **[Grid/SR]**
Person in your household	Age	Gender		Relationship to you
1.	_**[OPEN NUM 0-99]** yrs	1.M	2.F	Yourself
2.	_**[OPEN NUM 0-99]** yrs	1.M	2.F	1. Spouse / partner
				2. Child / step child
				3. Other relative
				98. Other (please specify_____)
…	_**[OPEN NUM 0-99]** yrs	1.M	2.F	1. Spouse / partner
				2. Child / step child
				3. Other relative
				98. Other (please specify_____)

[ASK ALL]

[Dummy variable 1 – do not display]

DV1 Number of school aged children (5 to 18) in household from A1_a **[SR]**

1. One school aged child (5 to 18)
2. Two or more school aged children (5 to 18)

99. None **[TERMINATE 4]**

[ASK ALL]
[If DV1=1 use Intro A, else use Intro B]
[If DV1=1 pre-fill grid with A2a=1 and A2b=1 (based on response at S1. Allow respondent to edit the pre-filled answer to A2a and A2b]
[Ask A2a, A2b for each child aged 5 to 18 in household] **[MANDATORY]**
[Only allow response at A2c where A2b=1]
[If response to A2c>14, display prompt: "Please confirm your response. According to administrative records, the quarantine period ran for 14 days]

A2 Intro A For how many days was your child who attended ^school^ asked to go into quarantine?
Intro B Of your children, please indicate which of them attended ^school^, which of them were asked to go into quarantine and how many days were they asked to go into quarantine for?

[Display for each member of the household aged 5 to 18 from A1]

<table>
<tr><th rowspan="3">Person in your household</th><th rowspan="3">Age</th><th rowspan="3">Gender</th><th colspan="2">a[GRID/SR]</th><th colspan="2">B [GRID/SR]</th><th>c [grid/OPEN NUM]</th></tr>
<tr><th colspan="2">Student at ^school^?</th><th colspan="2">Student asked to go into quarantine?</th><th rowspan="2">Days in quarantine</th></tr>
<tr><th>Yes</th><th>No</th><th>Yes</th><th>No</th></tr>
<tr><td></td><td></td><td></td><td>1</td><td>2</td><td>1</td><td>2</td><td>[OPEN NUM 1-14]</td></tr>
</table>

[IF all A2_a=2 or all A2_b=2 > TERMINATE 5]
[if no rows have A2_a=1 AND A2_a=1 then terminate 5] >because we need at least one child from that school who went into quarantine

[ASK ALL]
[Dummy variable 2 – do not display]
DV2 Number of school aged children in quarantine from A2b=1 **[SR]**

1. One child asked to go into quarantine
2. More than one child asked to go into quarantine
99. No children asked to go into quarantine **[TERMINATE 6]**

[ASK ALL]
[Dummy variable 2g – do not display]
DV2g Gender of DV2=1 child asked to go into quarantine **[SR]**

1. IF DV2=1 and gender of that child is Male **(A1b=1)**
2. IF DV2=1 and gender of that child is Female **(A1b=2)**
98. **All others (DV2=2)**

Module B: family members diagnosed with swine flu (between 22 May – 3 June 2009)

[ASK ALL]
[Display person number, age, gender of all household members from A1 in response grid]
B1 The following questions relate to household members who were diagnosed as having swine flu between 22nd May and 3rd June 2009. **[break]**
Which members of your household, if any, were diagnosed by a doctor or a laboratory as having swine flu? **[S/GRID/ROW]**

Person in your household	Age	Gender	Diagnosed as having swine flu	
			Yes	No
1			1	2
2			1	2

[Display following message if B1-1 or B1=2 not selected for any member of the household: "You don't appear to have answered for every member of your household. Please review your answers or click next to continue"]

[**ASK IF B1=1 to more than one row/person**: more than one person in household diagnosed with swine flu]
B2 Of those household member diagnosed with swine flu, who was diagnosed **first**?
Note: if more than one household member diagnosed with swine flu on the same day, select the youngest. **[SR]**
[GRID SHOW ONLY household members where B1=1]

Person in your household	Age	Gender	Who was diagnosed first
A1_1			☐ 1
A1_2			☐2
A1_3			☐3
A1_4			☐4
A1_5			☐5
A1_6			☐6
A1_7			☐7
A1_8			☐8
Don't know			☐

[Need to return **person number** person diagnosed first in data]

Module C: Tamiflu

[ASK ALL] [MANDATORY]

C1 Tamiflu is a medicine that was widely used during the swine flu outbreak to treat people who had swine flu and to stop people becoming infected.
Was Tamiflu prescribed for **anyone** in your household between 22nd May to 3rd June 2009? **[SR]**

1. Yes
2. No

[ASK IF C1=1 - someone in household prescribed Tamiflu] **[MANDATORY]**
[Display person number, age, gender of all household members from A1 in response grid]
C2 Which household members were prescribed Tamiflu? **[S/Grid/Row]**

Person in your household	Age	Gender	Prescribed Tamiflu	
			Yes	No
1			1	2
2			1	2

[BRING ERROR IF ALL C2_ = 2: Please select "Yes" next to the person in your household that was prescribed Tamiflu']

[ASK IF C1=1 - someone in household prescribed Tamiflu]
[ASKC3 and C4 in a loop for each C2=1 :member of household prescribed Tamiflu at C2]
C3 How many prescribed doses of Tamiflu were taken by…?

Family member: [Display gender of household member prescribed Tamiflu at C2], aged [Display age of household member prescribed Tamiflu at C2]

1. All doses
2. More than half of doses
3. Up to half of doses
97. Don't know
99. None

[ASK IF C3=2, 3, or 99 : person missed some or all doses of Tamiflu]
[If C3=2 or 3, use Intro A, else use Intro B]
C4 Intro A Why were some doses of Tamiflu missed?
Intro B Why were no prescribed doses of Tamiflu taken? **[MR]**

Family member: [gender], aged [age]

1. Got side effects **[display if C3=2 or 3**: took some Tamiflu]
2. Worried about side effects
3. Did not think was necessary
4. Forgot
5. Child would not take doses **[display if A1_c<19** :person prescribed Tamiflu at C2 is aged <19]
6. Did not receive medicine

98 Other (please specify___________)

Module D - quarantine measures

[ASK ALL] [D1A =MANDATORY, D1B NON MANDATORY]
[GRID SHOW all household members from A1]
[Pre-fill grid with responses for school aged children collected at A2b and A2c]
[Allow response in D1b where D1a=1]
[If response to D1b>14, display prompt: "Please confirm your response. According to administrative records, the quarantine period ran for no more than 14 days)]

D1 The next set of questions is about what families did during the quarantine period. A better understanding of family experiences can be used to improve quarantine recommendations in the future. **[break]**
Please answer the following questions as best you can.

Here is the information on the children in your household already gathered in the survey and below is/are the other people in your household
[show pre-filled grid with children info]

Which members of your household, other than school age children, were asked to go into quarantine? And for how many days was each person asked to go into quarantine?

			a **[S/Grid/Row]**		b **[S/OPEN NUM]**
Person in your household	Age	Gender	Asked to stay in quarantine?		Days in quarantine
			Yes	No	
1			1	2	**[OPEN NUM1-14]**
2			1	2	**[[OPEN NUM1-14]**

[ASK ALL] [MANDATORY]
D2 During the quarantine period, did quarantined members of your household make trips outside the home?

1. Yes
2. No

[ASK IF D2=1 – At least one quarantined member the household made a trip outside the home]
[GRID SHOW ALL members for which D1a=1]
D3 During the quarantine period, that is, from 22nd May to 3rd June, did **quarantined members** of your household visit these types of public places?

			a**[S/Grid/Row]**		b**[S/Grid/Row]**		c**[S/Grid/Row]**	
Quarantined person in your household	Age	Gender	An outdoor public space with no, or few people (e.g. taking a walk on beach, park lands)		An outdoor public space with lots of other people around (e.g. playground or market)		An enclosed public place, excluding doctor / hospital (e.g. movies, church, library, public transport)	
			Yes	No	Yes	No	Yes	No
			1	2	1	2	1	2

[ASK ALL]
[**LOOP** for each household member from D1 where D1a=1]
[Create response grid for each household member where the number of days (rows in grid) is equal to the total days in quarantine from D1b for that household member, else if d1b is blank (no data for that household member at D1b), create number of rows on grid based on the **highest number** of days in quarantine for any household member from D1b]
[Only display column option 3 (school) in grid if household member age is 5 to 18]
D4 To the best of your knowledge, where did **each member** of your household spend **most** of their time each day during the quarantine period?

[Display age and gender of household member:]
Family member: [gender], aged [age]

Day in quarantine	Family home	Friend or relative's home	School	Workplace outside home	Other place
1	1	2	3	4	98
2	1	2	3	4	98
3	1	2	3	4	98
4	1	2	3	4	98
5	1	2	3	4	98
6	1	2	3	4	98
7	1	2	3	4	98
8	1	2	3	4	98
9	1	2	3	4	98
11	1	2	3	4	98
12	1	2	3	4	98
13	1	2	3	4	98
14	1	2	3	4	98

[Repeat for all other household members]

[ASK ALL]
[Dummy variable 3 – do not display]
DV3 Number of days where most time spent **in family home** for any child 5 to 18 (e.g. if child 1= 5 days and child 2= 7 days, use 7 days)

Create numeric variable **[OPEN NUM] [0 -14]**

[ASK ALL]
[Dummy variable 4– do not display]
DV4 Number of children aged 5 to 18 where there was at least one day during the quarantine period where most of time spent each day was spent in the family home

1. One child
2. Two or more children
99. No children (aged 5 to 18 who spent most of time on at least one day during the quarantine period in the family home)

[ASK ALL]
[Dummy variable 5 – do not display]
DV5 Number of days where most time spent **outside** family home for any child 5 to 18

Create numeric variable 0 to 14

[ASK ALL]
[Dummy variable 6– do not display]

DV6 Number of children aged 5 to 18 where there was at least one day during the quarantine period where most of time spent each day was spent outside the family home

1. One child
2. Two or more children
99. No children (aged 5 to 18 who spent most of time on at least one day during the quarantine period outside the family home

[ASK IF DV3>0]
[If DV4=1, display "your child". If DV4=2, display "your children"]
D5 Thinking of the day(s) when (your child / your children) spent most of their time **in the family home**, to the best of your knowledge, on how many of those days, if any, did children from another household visit for **at least 15 minutes**?

Total days where (child / children) spent most of their time in the family home	Number of these days on which children from another household visited for at least 15 minutes
[display DV3 NUM answer]	__ days **[OPEN NUM] [0- DV3 ANSWER]**

[ASK IF DV5>0]
[If DV6=1, display "your child". If DV6=2, display "your children"]
[If DV2=2 display "they", if DV2g=1 display "he", if DV2g=2 display "she"]
D6 Thinking of the day(s) when (your child / your children) spent most of their time **outside the family home,** to the best of your knowledge, on how many of those days, if any, did (he / she / they) mix with children from another household for at least 15 minutes?

Total days where (child / children) spent most of their time outside family home	Number of these days on which (child / children) mixed with children from another household for at least 15 minutes
[display DV5 NUM answer]	__ days **[OPEN NUM] [0- DV5 ANSWER]**

[ASK ALL]
D7 During the quarantine period, did any adults other than household members come inside your home for more than 15 minutes? **[SR]**

1. Yes
2. No

Module E: your opinions about quarantine measures

Now we have some questions regarding your opinions on the quarantine measures

[ASK ALL]

E1 How would you rate….: **[**

S/Grid/Row]

	A minor burden		A moderate burden		A huge burden
Your family's **overall** experience with the quarantine measures	1	2	3	4	5

[ASK ALL]

E2 Thinking about what you knew at the time you experienced the quarantine measures, rather than what you think right now, would you say the length of time you were asked to stay in quarantine period was… **[S/R]**

1. Too short
2. About right
3. Too long

[ASK ALL]

E3 What did you think the consequences would be for you and your family of not following the quarantine measures? **[M/R]**

1. Nothing **[EXCLUSIVE]**
2. A fine
3. Arrest
98. Other (please specify_________)

[ASK ALL]

E4 To what extent do you agree with the following statements…
Please select one option for each row

[S/Grid/Row] [KEEP IN ORDER]

		Strongly disagree	Disagree	Neither agree nor disagree	Agree	Strongly agree
a	**At the time of the quarantine measures**, I understood what my family was being asked to do	1	2	3	4	5
b	**At the time of the quarantine measures**, I thought what we were being asked to do was reasonable given the circumstances	1	2	3	4	5
c	**Looking back now,** I think what we were asked to do was reasonable given the circumstances	1	2	3	4	5
d	In situations like this, people have a responsibility to do whatever it takes to control the spread of the disease	1	2	3	4	5

[ASK ALL]

Hypothetical situation
Imagine that an outbreak of another highly infectious disease were to occur in Australia in the future. The disease is new and not much is known about it yet, except that school children are especially at risk of catching this disease. The disease has caused several deaths overseas, but none yet in Australia.

E5 Given the circumstance described in the above situation, to what extent do you agree with the following statement….

		Strongly disagree	Disagree	Neither agree nor disagree	Agree	Strongly agree
a	I would be in favour of quarantine measures for **people diagnosed with the infection**	1	2	3	4	5

[ASK IF E5= 4 or 5]
[Display hypothetical situation **on same screen**]
E6 Would it change your view if you found out that people diagnosed with the infection……

	**[S/Grid/Row]**	Yes	No
a	Could be forced to comply with the quarantine measures	1	2
b	Would be quarantined in special facilities outside the home, such as an isolation unit in a hospital?	1	2

[ASK IF E5= 1 or 2 or 3]
[Display hypothetical situation **on same screen**]
E7 Would it change your view if you found out that:

	**[S/Grid/Row]**	Yes	No
a	Deaths had occurred in Australian children	1	2
b	A child from your child's school had died	1	2

[ASK ALL]
[Display hypothetical situation **on same screen**]
E8 Given the circumstance described in the above situation, to what extent do you agree with the following statement….

	**[S/Grid/Row]**	Strongly disagree	Disagree	Neither agree nor disagree	Agree	Strongly agree
a	I would be in favour of quarantine measures for people who are **not showing signs of infection** but who have had **close contact** with infected people	1	2	3	4	5

[ASK IF E8= 4 or 5]
[Display hypothetical situation **on same screen**]
E9 Would it change your view if you found out that people diagnosed with the infection……

	**[S/Grid/Row]**	Yes	No
a	Could be forced to comply with the quarantine measures	1	2
b	Would be quarantined in special facilities outside the home, such as an isolation unit in a hospital?	1	2

[ASK IF E8= 1 or 2 or 3]
[Display hypothetical situation **on same screen**]
E10 Would it change your view if you found out that:

	**[S/Grid/Row]**	Yes	No
a	Deaths had occurred in Australian children	1	2
b	A child from your child's school had died	1	2

[ASK ALL]
E11 If a child in your family became infected and had to be quarantined, would you prefer they were quarantined…

1. At home
2. In a separate facility, such as an isolation unit in a hospital

[ASK IF E11=2]
E12 Why would you prefer a separate facility? **[MR]**

1. Worried about other family members getting infected
2. Cannot get time off work to care for them
3. Too stressful at home
4. Feel that they would get better care/protection elsewhere

98. Other (please specify_________)

Module F: advice about quarantine

[ASK ALL]

[If DV2=1, display “child”, else display “children”]

F1 At the time of the swine flu outbreak in Victoria, information about the outbreak was coming from many sources. At the time your (child was / children were) asked to go into quarantine, where did you get information about what you were supposed to do in quarantine?

	**[S/Grid/Row]**	Yes	No
1	The Health Department	1	2
2	School	1	2
3	GP / other healthcare provider	1	2
4	Family / friends	1	2
5	Media (newspaper / tv)	1	2
98	Somewhere else (please specify__________)	1	2

[ASK IF AT LEAST ONE F1_ =1]

[For each source where F1_1 to F1_98=1]

[show grid where only F1_1 to F1_98=1 is displayed]

F2 Please rate how useful this information was….

	**[S/Grid/Row]**	Not at all useful 1	2	Somewhat useful 3	4	Extremely useful 5
1	The Health Department [display if F1_1=1]	1	2	3	4	5
2	School [display if F1_2=1]	1	2	3	4	5
3	GP / other healthcare provider [display if F1_3=1]	1	2	3	4	5
4	Family / friends [display if F1_4=1]	1	2	3	4	5
5	Media (newspaper / tv) [display if F1_5=1]	1	2	3	4	5
98	[display specified other from f1_98]<br>[display if F1_98=1]	1	2	3	4	5

Module G: school advice and support

[ASK ALL]
[If DV2=1, display "child", else display "children"]
[If DV2=2 display "their", if DV2g=1 display "his", if DV2g=2 display "her"]

G1 Schools played a central role during the time of the swine flu outbreak. The following questions will help us understand how useful school information was during this time.

To what extent do you agree with the following statements….

[S/Grid/Row]

		Strongly disagree	Disagree	Neither agree nor disagree	Agree	Strongly agree
a	When my (child's class room/ children's class rooms) closed, ^school^ provided me with clear information about what to do during the quarantine period.	1	2	3	4	5
b	^school^ provided me with good educational support, resources or materials for my (child / children) to continue (his / her / their) schooling during the quarantine period	1	2	3	4	5

[ASK ALL]
[If DV2=1, display "child", else display "children"]
G2 (Is your child / Are any of your children) in a Special Development School (SDS) or program?

1. Yes, SDS school
2. Yes, SDS program
3. No, not in SDS school or SDS program

Module H: care arrangements

[ASK ALL] [MANDATORY]
[LOOP for each child aged 5 to 18]
[If DV2=1, display "child", else display "children"]
H1 Who took care of your quarantined child described below during **school hours** for **half a day or more** during the quarantine period? (Select all that apply) **[MR]**

[Display age and gender of (first) school age child]
Family member: [gender], aged [age]

1. Looked after themselves
2. Parent(s) living in household
3. Other parent NOT living in household
4. Older brother or sister
5. Grandparent / other relative
6. Paid carer

98. Someone else (e.g. neighbour) (please specify____________)
[Repeat for other school age children]

[ASK ALL]
[Dummy variable 7A– do not display] **[SR]**
DV7A CHILD CARE STATUS, CREATE FOR EACH LOOP/CHILD AT H1

1. **IF H1≠2**
2. **IF DV7A≠1 AND 3 [EVERYONE WHO DO NOT FIT DV7A=1 OR DV7A=2 CONDITIONS]**
3. **IF H1=2 ONLY**

[ASK ALL]
[Dummy variable 7– do not display] **[SR]**
DV7 Parent caring status _**CAN CREATE FOR ALL H1 ANSWERS OR ALL DV7A ANSWERS**

1. **IF H1≠2 IN ALL LOOPED H1**
   (Exclusively other; parent did NOT look after any child at any time during quarantine period (H1 NOT 2 in any iteration))
2. **IF DV7≠1 AND 3 [EVERYONE WHO DO NOT FIT DV7=1 OR DV7=2 CONDITIONS]**
   (Mixed; mixed care arrangements, i.e. parent looked after one or more children for half a day or more during school hours at some stage during quarantine period (H1=2, for any school aged child), but other care arrangements were also used)
3. **[IF <u>H1=2 ONLY</u> IN ALL LOOPS AT H1]**
   (Exclusively parent; the only care arrangement for all children at H1 was parents living in household (H1=2 for all iterations))

[LOOP FOR EACH CHILD WHERE DV7A=2] (Ask for each child aged 5 to 18 at H1 with multiple care arrangements) [If DV2=1, display "child", else display "each of" and "children"]
H2 Please specify the approximate time spent in each type of care arrangement for (each of) your quarantined (child / children) during **school hours** during **the quarantine period**? **[S/Grid/ROW]**

Family member: [gender], aged [age] (Display age and gender of school age child with multiple care arrangements)
Total days in quarantine period**:** ^Display maximum quarantine days from D1^

**Care options [ONLY SHOW OPTIONS SELECTED IN H1]**	**1day**	**2 days**	**3 days**	**4 days**	**5 days**	**More than 5 days**
1. Looked after themselves	1	2	3	4	5	6
2. Parent(s) living in household	1	2	3	4	5	6
3. Other parent NOT living in household	1	2	3	4	5	6
4. Older brother or sister	1	2	3	4	5	6
5. Grandparent / other relative	1	2	3	4	5	6
6. Paid carer	1	2	3	4	5	6
98. **[H1 answer to 98]**	1	2	3	4	5	6

H2 – Revised version

Looked after themselves						
Parent(s) living in household						
Other parent NOT living in household						

Please select the nearest day option for each type of care used

Module J: work

[ASK ALL] [MANDATORY]
[IF AT LEAST ONE A1_c=1 (partner present in household) display intro A. Else display intro B]
[IF AT LEAST ONE A1_c=1 (partner present in household) display as two column response set, else as single column / "normal' question]
J1 Intro A: This section asks about your / your partner's work circumstances during the quarantine period.
At the time of the quarantine period, were you / your partner in paid employment of any kind?
Intro B: This section asks about your work circumstances during the quarantine period.
At the time of the quarantine period, were you in paid employment of any kind?

	a	b
**[S/grid/column]**	You	Your partner
Yes	1	1
No	2	2

[ASK IF J1a=1 or J1b=1]
[If J1a=1 and J1b=1, display "Did you / your partner"]
[If J1a=1 and (J1b=2 or J1b not asked), display "Did you.."]
[If J1b=1 and J1a=2, display "Did your partner…"]
[If J1a=1 and J1b=1, display as two column response set, else as single column / "normal' question]
J2 Did (you / your partner) have more than one paid job during the quarantine period?

	a	b
**[S/grid/column]**	You	Your partner
Yes	1	1
No	2	2

[ASK IF J2a=1 or J2b=1]
[If J2a=1 and J2b=1, display intro A]
[If J2a=1 and (J2b=2 or J2b not asked), display intro B]
[If J2b=1 and (J2a=1 or J2a not asked), display intro C]
PREJ3 Intro A You have indicated that you and your partner both had more than one job during the quarantine period. Please answer the following questions for your and your partner's MAIN job (the job with the most hours)
Intro B You have indicated that you had more than one job during the quarantine period. Please answer the following questions for your MAIN job during the quarantine period (the job with the most hours)
Intro C You have indicated that your partner had more than one job during the quarantine period. Please answer the following questions for your partner's MAIN job during the quarantine period (the job with the most hours)

[ASK IF J1a=1 or J1b=1]
[If J1a=1 and J1b=1, display "your / your partner's"]
[If J1a=1 and (J1b=2 or J1b not asked), display "your"]
[If J1b=1 and J1a=2, display "your partner's…"]
[If J1a=1 and J1b=1, display as two column response set, else as single column / "normal' question]

J3 What was (your / your partner's) paid job during the quarantine period? Please answer briefly in your own words:

**[OPEN/GRID/ROW]**	a<br>You	b<br>Your partner
1.Position title	**[OPEN CHA] [1-500]**	**[OPEN CHA] [1-500]**
2.Main tasks and duties	**[OPEN CHA] [1-500]**	**[OPEN CHA] [1-500]**

[ASK IF J1a=1 or J1b=1]
[If J1a=1 and J1b=1, display "your / your partner's"]
[If J1a=1 and (J1b=2 or J1b not asked), display "your"]
[If J1b=1 and J1a=2, display "your partner's…"]
[If J1a=1 and J1b=1, display as two column response set, else as single column / "normal' question]
J4 Was (your / your partner's) paid job full-time or part-time during the quarantine period?

	a	b
**[S/grid/column]**	You	Your partner
1. Full time hours (35 hours or more per week)	1	1
2. Part-time hours (less than 35 hours/week)	2	2

[ASK IF J1a=1 or J1b=1]
[If J1a=1 and J1b=1, display "your / your partner's"]
[If J1a=1 and (J1b=2 or J1b not asked), display "your"]
[If J1b=1 and J1a=2, display "your partner's…"]
[If J1a=1 and J1b=1, display as two column response set, else as single column / "normal' question]
J5 What was (your / your partner's) form of employment during the quarantine period?

	a	b
**[S/grid/column]**	You	Your partner
1. Permanent or on-going	1	1
2. Casual or Temporary	2	2
3. Fixed-term contract (i.e. employed for specific period of time)	3	3
4. Self-employed working by yourself	4	4
5. Self-employed and also employing others	5	5

[ASK IF J1a=1 or J1b=1]
[If J1a=1 and J1b=1, display "Were you / your partner"]
[If J1a=1 and (J1b=2 or J1b not asked), display "Were you"]
[If J1b=1 and J1a=2, display "Was your partner…"]
[If J1a=1 and J1b=1, display as two column response set, else as single column / "normal' question]
J6 (Were you / your partner / Were you / Was your partner) able to work from home during the quarantine period?

	a	b
**[S/grid/column]**	You	Your partner
Yes	1	1
No	2	2

[ASK IF J5a=1,2,3,5 or J5b=1,2,3,5]
[If J5a=1,2,3,5 and J5b=1,2,3,5, display "your / your partner's"]
[If J5a=1,2,3,5 and (J5b=4 or J5b not asked), display "your"]
[If J5b=1,2,3,5 and J5a=4, display "your partner's…"]
[If J5a=1,2,3,5 and J5b=1,2,3,5, display as two column response set, else as single column / "normal' question]
J7 Did your / your partner's job provide access to any of the following type of leave?

**[3D grid]**	a		b	
	You		Your partner	
1. Sick leave	1. Yes	2. No	1. Yes	2. No
2. Paid carer's leave	1. Yes	2. No	1. Yes	2. No
3. Annual leave	1. Yes	2. No	1. Yes	2. No

Add don't know option for each
[Display following message if any section of question not answered Please review your answers or click next to continue]

[ASK IF J1a=1 or J1b=1]
[If J1a=1 and J1b=1, display "you / your partner"]
[If J1a=1 and (J1b=2 or J1b not asked), display "you"]
[If J1b=1 and J1a=2, display "your partner…"]
[If J1a=1 and J1b=1, display as two column response set, else as single column / "normal' question]
J8. Did you think that (you / your partner) could take unpaid leave for up to a week without affecting your job security?

	a	b
**[S/grid/column]**	You	Your partner
Yes	1	1
No	2	2
Don't know	3	3

[Display following message if any section of question not answered Please review your answers or click next to continue]

Module - work and childcare arrangements

[ASK IF DV7=1 or DV7=2 – care arrangement other than parent during quarantine period]
[If DV2=1 use "child", else use "children"]
[If DV2=2 use "themselves"; if DV2g=1 use "himself"; if DV2g=2 use "herself"]

K1 Earlier you indicated that someone other than a parent took care of your quarantined (child / children) for half a day or more during the quarantine period. **[break]** Your responses to the following questions will help us understand how working conditions may affect care arrangements for children.

On the day / days when a parent was not primarily looking after your (child / children) for a half day or more, was this **mainly** because of: **[SR]**

1. Parental work
2. Parental study or training
3. (Child / children) able to look after (himself / herself / themselves) **[display if in ANY LOOP H1=1** (any iteration) : child looking after self]]

98. Other reason (please specify_________)

[ASK IF K1=1 – Parent could not care for child due to work]

K2 If a parent could not stay home due to work, was this *mainly* because they did not have **paid leave** entitlements? **[SR]**

1. Yes
2. No

[ASK IF K2=1 – Parent did not have paid leave entitlements]

K3 Did they not have paid leave because….**[SR]**

1. Paid leave was not provided in that job
2. Paid leave already used up

[ASK IF K1=1 or K1=3]
[If DV2=1 use "child", else use "children"]

K4 Which of the following factors, if any, were important in making your care arrangements for your quarantined (child / children) during the quarantine period? (Select all that apply) **[MR]**

1. Suitable arrangements could be made without needing to use leave
2. My (child was / children were) fine, no special arrangements were necessary
3. Health risks were not serious enough
4. Fear of losing job
5. Work commitments (too much work to do, no one to cover tasks at work, etc)

98. Other (please specify_________)

[ASK IF K1=1 or K1=3]
[IF ONLY 1 SELECTED AT K4, AUTOPUNCH WITHOUT ASKING K5]
[If DV2=1 use "child", else use "children"]
[Display responses to K4]

K5 Which **one** of these was **the most important factor** in making your care arrangements for your quarantined (child / children) during the quarantine period? **[SR]**

1. Suitable arrangements could be made without needing to use leave
2. My (child was / children were) fine, no special arrangements were necessary

3. Health risks were not serious enough
4. Fear of losing job
5. Work commitments (too much work to do, no one to cover tasks at work, etc)
98. **^display answer 98 at K4 if answered^**

[ASK IF DV7=2 or DV7=3 AND J1a=1 or J1b=1] – parent provided some care during quarantine period and parent was working]
[If DV2=1 use "child", else use "children"]
K6 Earlier you indicated that a parent provided care during school hours in the quarantine period. When a parent provided care for your (child / children) during school hours during the period of quarantine, did the parent take time off work to provide care? **[SR]**

1. Yes
2. No

[ASK IF K6=1 – took time off work]
[If DV2=1 use "child", else use "children"]
K7 Did the parent lose pay to care for your (child / children)? **[SR]**

1. Yes
2. No

[ASK IF K7=1 – lost pay]
K8 Why did the parent lose pay? **[SR]**

1. Paid leave not provided by the ~~my~~ job
2. Paid leave already used up
3. Lost the job for taking take time off

[ASK IF K7=1 – lost pay]
K9 Which of the following, if any, happened because pay was lost?

	**[S/grid/row]**	Yes	No
a	Had difficulty paying a bill	1	2
b	Had difficulty paying the mortgage or rent	1	2
c	Had to borrow money	1	2
d	Other financial problems	1	2

[ASK IF J1a=1 or J1b=1 – respondent and / or partner work]
[If DV1=1 use "child", else use "children"]
K10 In the 12 months prior to the swine flu outbreak, did your (child / children) ever need to stay home from school? **[SR]**

1. Yes
2. No

[ASK IF K10=1 – child / children needed to stay home]
[If DV1=1 use “child”, else use “children”]
K11 Which, if any, of the following care options have you used in the past 12 months when your (child has / children have) needed to stay home from school? (select all that apply) **[MR]**

1. I took the day off work [display if J1a=1]
2. My partner took the day off work [display if J1b=1]
3. Grandparents / other family took care of the (child / children]
4. A friend / neighbour took care of the (child / children]
5. Paid care in home
6. Paid care in another location
7. I worked from home [display if J1a=1]
8. My partner worked from home [display if J1b=1]
9. Parent not working that day, would look after them
98. Other (please specify_________)
99. None of these

[ASK IF K10=2 – child / children did not need to stay home]
[If DV1=1 use “child”, else use “children”]
K12 Which, if any, of the following child care options **would** you use if your (child / children) needed to stay home from school? (select all that apply) **[MR]**

1. I would take the day off work [display if J1a=1]
2. My partner would take the day off work [display if J1b=1]
3. Grandparents / other family would take care of the (child / children]
4. A friend / neighbour would take care of the (child / children]
5. Paid care in home
6. Paid care in another location
7. I would work from home [display if J1a=1]
8. My partner would work from home [display if J1b=1]
9. Parent not working that day, would look after them
98. Other (please specify_________)
99. None of these

Module L: education

[ASK ALL]
[IF AT LEAST ONE A1_c=1 (partner present in household) display intro A. Else display intro B]
[IF AT LEAST ONE A1_c=1 (partner present in household) display as two column response set, else as single column / "normal' question]
L1 Intro A What is the highest year of primary or secondary school you / your partner have **completed**?
Intro B What is the highest year of primary or secondary school you have **completed**?

**[S/grid/column]**	a<br>You	b<br>Your partner
Did not go to school	1	1
Year 8 or below	2	2
Year 9 or equivalent	3	3
Year 10 or equivalent	4	4
Year 11 or equivalent	5	5
Year 12 or equivalent	6	6

[ASK ALL]
[IF AT LEAST ONE A1_c=1 (partner present in household) display intro A. Else display intro B]
[IF AT LEAST ONE A1_c=1 (partner present in household) display as two column response set, else as single column / "normal' question]
L2 Intro A Since leaving school have you / your partner **completed** a trade certificate or any other educational qualification?
Intro B Since leaving school have you **completed** a trade certificate or any other educational qualification?

**[S/grid/column]**	a<br>You	b<br>Your partner
Yes	1	1
No	2	2

[ASK IF L2a=1 or L2b=1]
[If L2a=1 and L2b=1, display “you / your partner”]
[If L2a=1 and (L2b=2 or L2b not asked), display “you..”]
[If L2b=1 and L2a=2, display “your partner…”]
[If L2a=1 and L2b=1, display as two column response set, else as single column / “normal’ question]
L3 What is the level of the *highest* qualification (you / your partner) have **completed** since leaving high school?
If (you / your partner) are currently undertaking a course please tick your highest completed qualification.

	a	b
**[S/grid/column]**	You	Your partner
Trade or business certificate	1	1
Apprenticeship	2	2
Associate diploma	3	3
Undergraduate diploma	4	4
Bachelor degree	5	5
Post graduate diploma	6	6
Masters degree	7	7
Doctorate	8	8
Not Sure	9	9
Other (please specify________________)	98	98

Module M: home and living circumstances

[ASK ALL]

M1 This section asks you about your home and usual living circumstances. These answers help us to understand how quarantine may affect different types of households. **[SR]**

So firstly, is your dwelling…

1. Owned outright
2. Owned with a mortgage
3. Being purchased under a rent/buy scheme
4. Being rented
5. Being occupied rent free

98. Other (please specify________)

[ASK IF M1=4]

M2 Who is it being rented from? **[SR]**

1. Real estate agent
2. Government Housing – Office of Housing Victoria
3. Parent / Other relative not in this dwelling
4. Other person not in this dwelling
5. Employer - Government (including Defence Housing Authority)
6. Employer - Private
7. Housing co-operative; Community or Church Group

[ASK ALL]

M3 Which of these categories contains the combined income of everyone in your household (before tax and other deductions are taken out), during the last financial year (1 July 2008 to 30 June 2009)? **[SR]**

Please include income from all sources

1. $2,000 or more per week ($104,000 or more per year)
2. $1,600 - $1,999 per week ($83,200 - $103,999 per year)
3. $1,300 - $1,599 per week ($67,600 - $83,199 per year)
4. $1,000 - $1,299 per week ($52,000 - $67,599 per year)
5. $800 - $999 per week ($41,600 - $51,999 per year)
6. $600 - $799 per week ($31,200 - $41,599 per year)
7. $400 - $599 per week ($20,800 - $31,199 per year)

8 $1 - $399 per week ($1 - $20,799 per year)

99. Nil income

[ASK ALL]

M4 Suppose you had only one week to raise $2000 for an emergency. Which of the following best describes how hard it would be for you to get that money? **[SR]**

1. Could easily raise $2000
2. Could raise $2000, but it would involve some sacrifices
3. Would have to do something drastic to raise $2000
4. Couldn't raise $2000

Module N: general comments from respondents

[ASK ALL]

N1 Based on your experience with quarantine, if another outbreak meant that quarantine was necessary, do you have any suggestions for improving how these measures should be introduced in the future? **[SR]**

1. Yes (please specify____________)
2. No

[INSERT COMPLETE SCRIPT HERE, so all respondents who click "Next after N1 are "completes"]

[ASK ALL – COMPLETE PAGE]

You have now completed the survey and submitted your responses.

Thank you very much for your participation, you may now close this window or click "Next" and you will be redirected to the Home page of the University of Melbourne.

[redirect to respondents to http://www.sph.unimelb.edu.au/]

>> *it's important that the respondent is shown clearly that their responses to the survey have been submitted.*

END OF QUESTIONNAIRE

THANK YOU FOR YOUR PARTICIPATION

Termination location	Termination script to display at screen out page
TERMINATE 1	Thank you for your interest, but this study is focused on children who were asked to go into quarantine at (school)
TERMINATE 2	Thank you for your interest, but this study is focused on children who were asked to go into quarantine.<br><br>This questionnaire is only for parents whose children were asked to go into quarantine, thank you for your time.
TERMINATE 3	Thank you for your interest, but this study is focused on parents of children who were asked to go into quarantine.
TERMINATE 4	Thank you for your interest, but this study is focused on parents of children who were asked to go into quarantine.
TERMINATE 5	Thank you for your interest, but this study is focused on children who were asked to go into quarantine at (school)
TERMINATE 6	Thank you for your interest, but this study is focused on children who were asked to go into quarantine.

[ALL]

Completion_status: DumVariable_record status of respondent according to SRC definition **[SR]**

1 Complete **[COMPLETE WITH IP ≠ 150.101.220.138 and 150.101.167.202]**
2 SRC 1800 administered completion **[COMPLETE WITH IP = 150.101.220.138 OR 150.101.167.202]**
3 Partially complete **[INCOMPLETE]**
4 Disqualified **[SCREEN OUTS]**

[IF Completion_status=4]
Termpoint : DumVariable_ record at which stage respondents screened out

1 TERM 1 **[SCREEN_IND=1]**
2 TERM 2 **[SCREEN_IND=2]**
3 TERM 3 **[SCREEN_IND=3]**
4 TERM 4 **[SCREEN_IND=4]**
5 TERM 5 **[SCREEN_IND=5]**
6 TERM 6 **[SCREEN_IND=6]**
